# Spatiotemporal models for predicting high pollen concentration level of *Corylus*, *Alnus*, and *Betula*

**DOI:** 10.1007/s00484-015-1077-8

**Published:** 2015-10-21

**Authors:** Jakub Nowosad

**Affiliations:** Institute of Geoecology and Geoinformation, Adam Mickiewicz University, Dzięgielowa 27, 61-680 Poznań, Poland

**Keywords:** Allergenic pollen, Betulaceae, Predictive modeling, Spatiotemporal models, Machine learning, Random forest

## Abstract

*Corylus*, *Alnus*, and *Betula* trees are among the most important sources of allergic pollen in the temperate zone of the Northern Hemisphere and have a large impact on the quality of life and productivity of allergy sufferers. Therefore, it is important to predict high pollen concentrations, both in time and space. The aim of this study was to create and evaluate spatiotemporal models for predicting high *Corylus*, *Alnus*, and *Betula* pollen concentration levels, based on gridded meteorological data. Aerobiological monitoring was carried out in 11 cities in Poland and gathered, depending on the site, between 2 and 16 years of measurements. According to the first allergy symptoms during exposure, a high pollen count level was established for each taxon. An optimizing probability threshold technique was used for mitigation of the problem of imbalance in the pollen concentration levels. For each taxon, the model was built using a random forest method. The study revealed the possibility of moderately reliable prediction of *Corylus* and highly reliable prediction of *Alnus* and *Betula* high pollen concentration levels, using preprocessed gridded meteorological data. Cumulative growing degree days and potential evaporation proved to be two of the most important predictor variables in the models. The final models predicted not only for single locations but also for continuous areas. Furthermore, the proposed modeling framework could be used to predict high pollen concentrations of *Corylus*, *Alnus*, *Betula*, and other taxa, and in other countries.

## Introduction

*Corylus* L. (hazel), *Alnus* Mill. (alder), and *Betula* L. (birch) are considered to be among the most important sources of allergic pollen in the temperate zone of the Northern Hemisphere (D’Amato et al. 2007). According to Heinzerling et al. (2009), approximately 21–24 % of Europeans are sensitized to tree pollen from the Betulaceae family. These rates in Poland are 22.3, 22.8, and 27.7 %, respectively, for *Corylus*, *Alnus*, and *Betula* (Heinzerling et al. 2009). There are also high levels of cross-reactivity between *Corylus*, *Alnus*, and *Betula* (Ebner et al. 1995). As a consequence, *Corylus* and *Alnus* pollination can lead to more marked clinical symptoms during a *Betula* pollen season (D’Amato et al. 2007).

Pollen concentration in the air is the resultant of many factors of different temporal and spatial variability. The spatial distribution of the taxa and phytosociological and habitat relationships mainly affect the temporal variability and intensity of pollen seasons. Moreover, meteorological factors have an impact not only on the production and release but also on the dispersal of tree pollen grains. Previous studies found a relationship between the temperature in the preceding year and the annual pollen sum (Latałowa et al. 2002; Rasmussen 2002). The influence of air temperature on pollen concentration has often been reported (Rodríguez-Rajo et al. 2004; Puc^2007^, ^2012^; Kizilpinar et al.^2011^). The impact of other meteorological parameters, such as precipitation, wind speed, and humidity, has also been reported (Latałowa et al. 2002; Puc^2007^, ^2012^). In addition, recent studies have shown that the temporal variations in *Corylus*, *Alnus*, and *Betula* pollen counts are related to three groups of factors. The temporal span of these factors are (i) daily, (ii) approximately 3.5 days, and (iii) more than 15 days (Nowosad et al. 2015).

Spatial analyses in aerobiology mainly involve the following: the comparison between two or more different localizations (Stach et al. 2008; Puc and Kasprzyk 2013; Sauliene et al. 2014); the description of spatial variation of pollen season properties or pollen concentrations (Emberlin et al. 2002; Rieux et al. 2008; Myszkowska et al. 2010; Nowosad et al. 2015); or the investigation of pollen transportation using back trajectories (Skjoth et al.^2008^, ^2009^; Veriankaite et al.^2009^; Rojo and Pérez-Badia 2015). There have been only a few studies in which spatial models of *Betula* pollen count were built. Vogel et al. (2008) included the parametrisation of the emissions of *Betula* pollen into a non-hydrostatic mesoscale model. Sofiev et al. (2013) used the SILAM dispersion model to create a *Betula* pollen emission model. According to the author’s knowledge, spatial models of *Corylus* and *Alnus* pollen concentration have not been reported.

The main aim of this study was to develop spatiotemporal predictive models of *Corylus*, *Alnus*, and *Betula* pollen concentration levels, using preprocessed gridded meteorological data. Based on the final models, it is possible to predict pollen concentration levels, not only in aerobiological monitoring sites but also at unsampled locations.

## Materials and methods

**Fig. 1:**
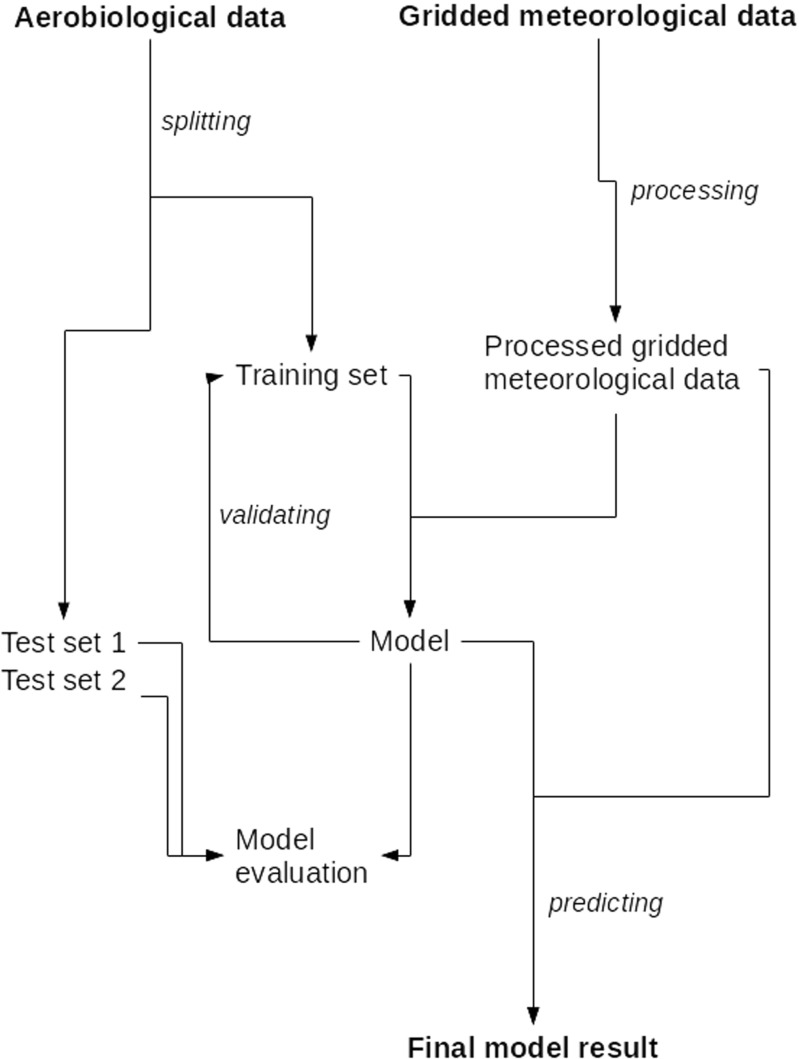
A flowchart of the processes for the predictive mapping of the pollen concentration levels

The development of spatiotemporal predictive models of *Corylus*, *Alnus*, and *Betula* pollen concentration levels was a main goal of this study. For each taxon, the workflow was as follows. Aerobiological data were split into a training set and two test sets, whilst gridded meteorological data were preprocessed. Using data from the training set, optimal model parameters were estimated. Final model performance was obtained by comparison of model prediction with true pollen concentration levels from both of the test sets. Afterwards, the final model and processed gridded meteorological data were used to create spatiotemporal predictions for every available day for each grid cell in the study area (Fig. 1). All the calculations were carried out using R (R Core Team 2014) and R packages (Liaw and Wiener 2002; Pebesma and Bivand 2005; Wickham 2009; Kuhn 2015). The workflow is described in detail in the subsections below.

### Aerobiological data

The monitoring of the concentrations of *Corylus*, *Alnus*, and *Betula* pollen was conducted in 11 cities in Poland: Bydgoszcz, Gdańsk, Kraków, Lublin, Łódź, Olsztyn, Poznań, Rzeszów, Siedlce, Sosnowiec, and Szczecin. The aerobiological studies covered between 2 (Siedlce) and 16 (Poznań) years of measurements (Table 1).Daily pollen concentrations were measured using the recommendations of the European Aerobiology Society’s Working Group on Quality Control (Galán et al. 2014).

**Table 1:** Population and area of the cities with the aerobiological monitoring sites; longitude, latitude, altitude, and the studied years of the aerobiological monitoring sites

Site	Population (in thousands)	Area (*k* *m* ^2^)	*λ* (DD)	*ϕ* (DD)	Altitude (a.s.l.)	Studied years
Bydgoszcz	361	176	18.13	53.14	51	2009–2011
Gdańsk	460	262	18.61	54.39	12	1998–2005, 2009–2011
Kraków	758	327	19.96	50.06	207	1998–2005, 2009–2011
Łódź	719	293	19.47	51.77	216	2003–2005, 2009–2011
Lublin	348	147	22.54	51.24	194	2001–2005, 2009–2011
Olsztyn	175	88	20.49	53.78	132	2009–2011
Poznań	551	262	16.92	52.47	93	1996–2011
Rzeszów	182	116	22.02	50.03	201	1997–2005, 2009–2011
Siedlce	76	32	22.31	52.18	147	2010–2011
Sosnowiec	214	91	19.14	50.30	253	2001–2011
Szczecin	409	301	14.55	53.44	28	2002–2011

**Fig. 2:**
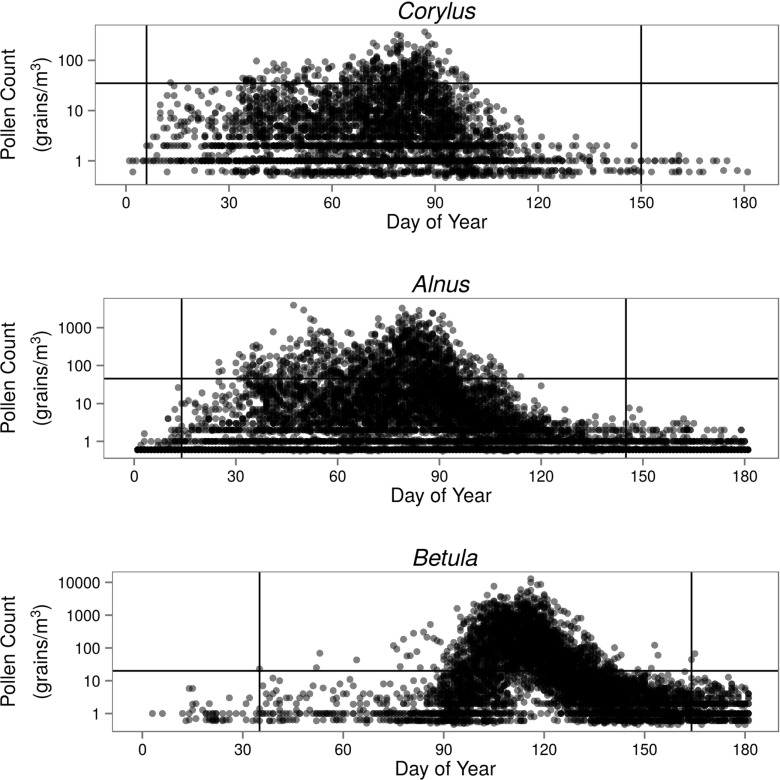
Pollen count of *Corylus*, *Alnus*, and *Betula* for all of the analyzed sites and all years, on a logarithmic scale. *Vertical lines* indicate the temporal scope of analysis for each taxon. *Horizontal lines* separate the two pollen concentration levels of low and high: 35 grains/m ^3^ for *Corylus*, 45 grains/m ^3^ for *Alnus*, and 20 grains/m ^3^ for *Betula*

The *Corylus*, *Alnus*, and *Betula* pollen season limits at each location, and each year was calculated using the 99 % method. According to this method, the onset of the pollen season was determined when 0.5 % of the total annual pollen count was noted, whereas the end of the season was determined when 99.5 % of pollen grains were recorded. The maximum range of the *Corylus* pollen season, using all the data, was between 6 and 150 days of the year; for *Alnus*, the number of days ranged from 14 to 145 days; for *Betula*, from 35 to 164 days. Models were built and evaluated using the data from these periods (Fig. 2).

Based on first symptom values for patients allergic to each taxon, two levels of concentration (low and high) were distinguished (Rapiejko et al. 2007). The limits were set at 35 grains/m ^3^ for *Corylus*, 45 grains/m ^3^ for *Alnus*, and 20 grains/m ^3^ for *Betula* (Fig. 2).

### Grid data

AGRI4CAST Interpolated Meteorological Data (Baruth et al. 2007) were used as the main input data. The AGRI4CAST database is a collection of daily meteorological parameters from weather stations interpolated to a 25 × 25 km grid and contains data from 1975 to 2014. For the purpose of this study, grid data were restricted to the area of Poland and a zone of 200 km around Polish borders. The buffer value was based on the longest distance between the nearest aerobiological sites (Szczecin and Poznań): approximately 200 km.

### Data split

The models were designed to predict pollen concentration levels (i) in the aerobiological monitoring sites, and (ii) in sites without aerobiological monitoring. For this purpose, data were split into the following three sets:

- Training set, which contained 2/3 of the data from eight cities in Poland (Gdańsk, Kraków, Lublin, Olsztyn, Poznań, Rzeszów, Siedlce, and Szczecin). The data were split randomly based on the dates available in this study.
- First test set, which contained the remaining 1/3 of the data from the same eight cities (Gdańsk, Kraków, Lublin, Olsztyn, Poznań, Rzeszów, Siedlce, and Szczecin).
- Second test set, which contained data from Bydgoszcz, Łódź, and Sosnowiec.

### Predictor variables

The pollen concentration level at each site and on each day from the training set was used as an outcome variable. The daily meteorological variables were preprocessed. Afterwards, preprocessed meteorological variables from the grid cells corresponding to the location of aerobiological sites were selected as predictor variables (Table 2):

- The average monthly temperatures for each month over the previous year for each site
- Four- and 16-day averages, calculated for each of the meteorological parameters. The temporal span of these factors was based on a recent study which showed that the temporal variations in *Corylus*, *Alnus*, and *Betula* pollen counts are related to factors that change (i) diurnally, (ii) approximately every 3.5 days, and (iii) in more than 15 days (Nowosad et al. 2015). These values were then lagged by 1 day
- Cumulated growing degree days (GDD), lagged by 1 day
- Longitude, latitude, and altitude of grid cell

**Table 2:** Explanation of the predictor variable abbreviations used in spatiotemporal modeling of *Corylus*, *Alnus*, and *Betula* pollen concentration levels

Abbreviation	Predictor variable name	Unit
TAVG_JANUARY_PREVYEAR	Average monthly temperature for January in the preceding year	^∘^C
TAVG_FEBRUARY_PREVYEAR	Average monthly temperature for February in the preceding year	^∘^C
TAVG_MARCH_PREVYEAR	Average monthly temperature for March in the preceding year	^∘^C
TAVG_APRIL_PREVYEAR	Average monthly temperature for April in the preceding year	^∘^C
TAVG_MAY_PREVYEAR	Average monthly temperature for May in the preceding year	^∘^C
TAVG_JUNE_PREVYEAR	Average monthly temperature for June in the preceding year	^∘^C
TAVG_JULY_PREVYEAR	Average monthly temperature for July in the preceding year	^∘^C
TAVG_AUGUST_PREVYEAR	Average monthly temperature for August in the preceding year	^∘^C
TAVG_SEPTEMBER_PREVYEAR	Average monthly temperature for September in the preceding year	^∘^C
TAVG_OCTOBER_PREVYEAR	Average monthly temperature for October in the preceding year	^∘^C
TAVG_NOVEMBER_PREVYEAR	Average monthly temperature for November in the preceding year	^∘^C
TAVG_DECEMBER_PREVYEAR	Average monthly temperature for December in the preceding year	^∘^C
TMAX_4DAYS_AVG_1DAYLAG	Average maximum temperature in preceding 4 days	^∘^C
TMAX_16DAYS_AVG_1DAYLAG	Average maximum temperature in preceding 16 days	^∘^C
TMIN_4DAYS_AVG_1DAYLAG	Average minimum temperature in preceding 4 days	^∘^C
TMIN_16DAYS_AVG_1DAYLAG	Average minimum temperature in preceding 16 days	^∘^C
VAPORPRESSURE_4DAYS_AVG_1DAYLAG	Average vapor pressure in preceding 4 days	hPa
VAPORPRESSURE_16DAYS_AVG_1DAYLAG	Average vapor pressure in preceding 16 days	hPa
WINDSPEED_4DAYS_AVG_1DAYLAG	Average wind speed in preceding 4 days	m/s
WINDSPEED_16DAYS_AVG_1DAYLAG	Average wind speed in preceding 16 days	m/s
PRECIPITATION_4DAYS_AVG_1DAYLAG	Average daily precipitation in the preceiding 4 days	mm
PRECIPITATION_16DAYS_AVG_1DAYLAG	Average daily precipitation in the preceiding 16 days	mm
EVAPORATION_4DAYS_AVG_1DAYLAG	Average potential evaporation in the preceding 4 days	mm/day
EVAPORATION_16DAYS_AVG_1DAYLAG	Average potential evaporation in the preceding 16 days	mm/day
RADIATION_4DAYS_AVG_1DAYLAG	Average total global radiation in the preceding 4 days	KJ/m ^2^/day
RADIATION_16DAYS_AVG_1DAYLAG	Average total global radiation in the preceding 16 days	KJ/m ^2^/day
GDD_1DAYLAG	Cummulated growing degree days (GDD) lagged by one day	GDD
LONGITUDE	Grid cell longitude	degrees
LATITUDE	Grid cell latitude	degrees
ALTITUDE	Average altitude of grid cell	m a.s.l.

For each day and each grid cell, growing degree days (GDD) were calculated as follows:

1 $$ \text{Daily GDD} = \frac{T_{\textnormal{max}}+T_{\textnormal{min}}}{2}-T_{\text{base}} $$

where *T*_max_ is the daily maximum temperature, *T*_min_ is the daily minimum temperature, and *T*_base_ is the base temperature.

GDDs were accumulated by adding the number of degree days that accumulated each day from January 1. The base temperature was designated as 5 ^∘^C, which is the standard threshold temperature for growth in temperate species (Dahl et al. 2013). If the daily maximum temperature is not higher than the base temperature, then no degree days accumulate.

#### Model development

Random forest (Breiman 2001) was used to spatiotemporally predict the pollen level of *Corylus*, *Alnus*, and *Betula*. For classification tasks, it is an ensemble of unpruned classification trees. The prediction is made by aggregating the prediction of the ensemble. The random forest algorithm uses two parameters: ntree (the number of trees) and mtry (the number of input variables randomly chosen at each split). In this study, ntree was set to 500, while optimal values of mtry were obtained by using 100 repetitions of ten-fold cross-validation on the training set.

**Fig. 3:**
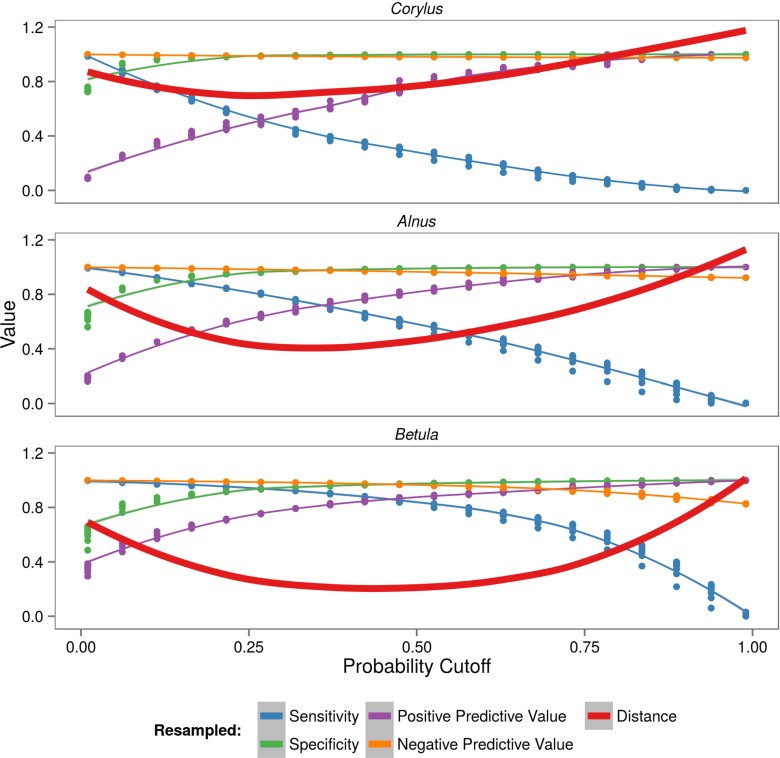
Resampled values of sensitivity, specificity, positive predictive value, negative predictive value, and the numerical distance between those values for each taxon model

The random forest algorithm focuses on overall accuracy and, consequently, does not work well for imbalanced data. The *Corylus*, *Alnus*, and *Betula* pollen concentration levels were highly imbalanced. In the period analyzed, the proportion between high and low levels was 330 to 13,182 for *Corylus*, 966 to 11,348 for *Alnus*, and 2104 to 9933 for *Betula*. In this study, an optimizing probability threshold technique was applied (Kuhn and Johnson 2013). This approach determines alternative cutoffs for the predicted probabilities. Using resampling, 20 different threshold values were tried on the training sets. Optimal threshold values were obtained by minimizing the distance between obtained sensitivity (Sens), specificity (Spec), positive predictive value (Ppv), negative predictive value (Npv), and the best possible performance (Fig. 3). In all cases, the best possible performance was equal to 1.

2 $$ \text{Distance}=\sqrt{ \begin{array}{l} (1 - \text{Sens})^{2} + (1 - \text{Spec})^{2}\\ \hspace*{11pt}+ (1 - \text{Ppv})^{2} + (1 - \text{Npv})^{2} \end{array}} $$

A permutation importance (mean decrease in accuracy) was used to determine input variable importance (Breiman 2001; Liaw and Wiener 2002).

#### Evaluation of the models performance

The accuracy of a model is not an appropriate measure of performance of prediction with highly imbalanced data. Instead, Kappa statistic, sensitivity, specificity, positive predictive value, and negative predictive value were used to evaluate the performance of the models:

3 $$ \text{Kappa} = \frac{O - E}{1 - E} $$

where *O* is the observed accuracy, and *E* is the accuracy expected to be achieved based on the marginal totals of the confusion matrix. The Kappa statistic values range from -1 to 1. A value of 1 indicates perfect agreement between the observed and predicted classes; a value of 0 indicates no agreement; negative values indicate that the predicted class is the opposite of the reference class (Kuhn and Johnson 2013).

4 $$ \text{Sensitivity} = \frac{TP}{TP + FN} $$

5 $$ \text{Specificity} = \frac{TN}{TN + FP} $$

6 $$ \text{Positive predictive value} = \frac{TP}{TP + FP} $$

7 $$ \text{Negative predictive value} = \frac{TN}{TN + FN} $$

where *TP* are the true positives (high levels predicted correctly), *FP* are false positives (high levels incorrectly predicted), *TN* are true negatives (low levels correctly predicted), and *FN* are false negatives (low levels incorrectly predicted).

*Corylus*, *Alnus*, and *Betula* models were evaluated on two test sets. Firstly, the temporal modelsŠ performance was determined by comparison between true pollen concentration levels and predictions on the first test set. Secondly, models’ predictions were compared with true pollen concentration levels from Bydgoszcz, Łódź, and Sosnowiec (the second test set). Data from these cities were not used for model creation. Thus, the evaluation was used to determinate spatial quality of the models.

## Results

### Probability threshold

For each taxon, the final model had different probability thresholds. In the *Corylus* models, the probability threshold dividing low and high pollen concentration levels was optimized to 0.22. In the other taxa, the class imbalance was less severe, and thus the optimal probability threshold value was higher: 0.32 for *Alnus* and 0.42 for *Betula* (Fig. 3).

### Variable importance

**Fig. 4:**
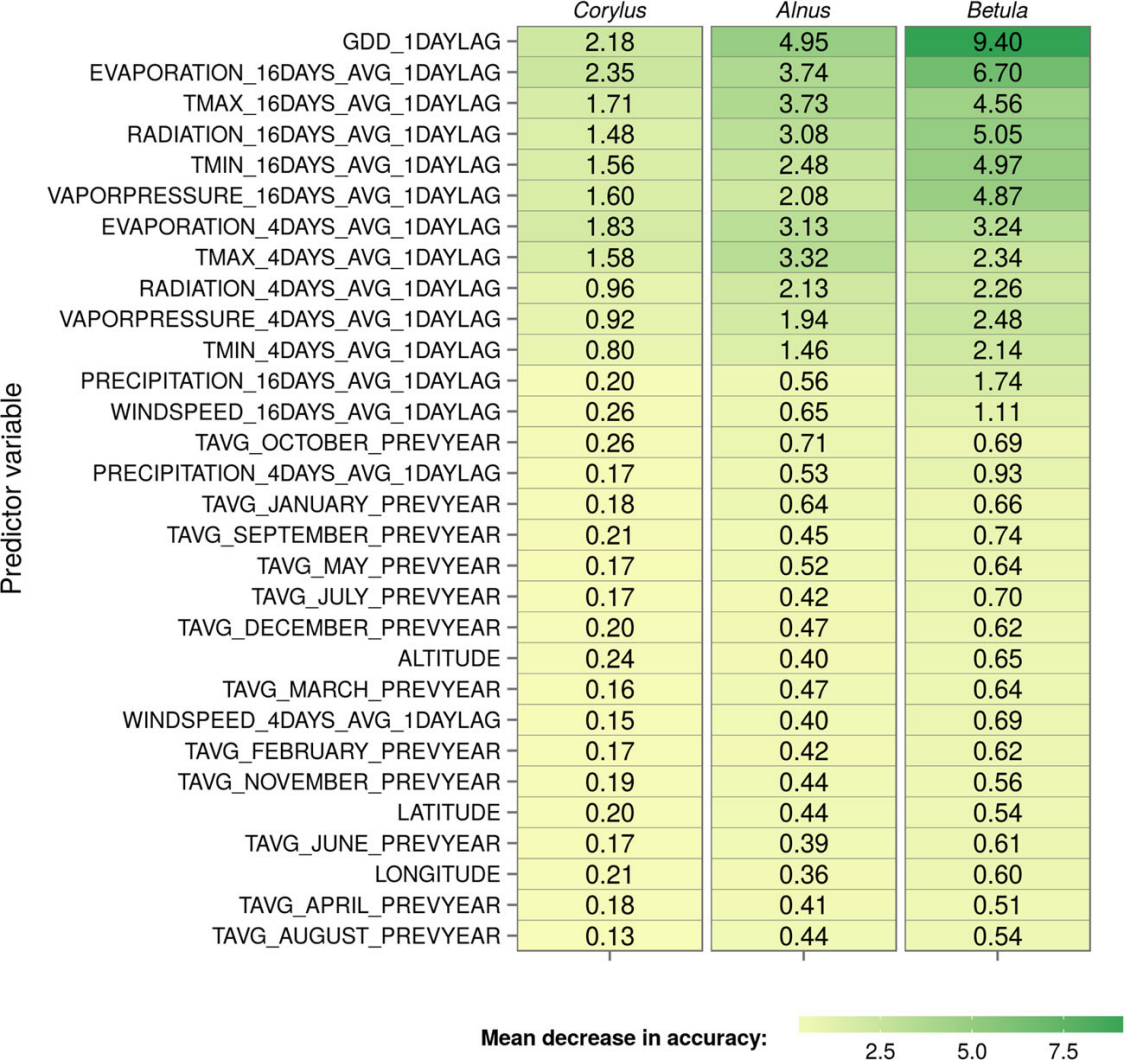
Variable importance of each input variable for *Corylus*, *Alnus*, and *Betula* models. The variables are showed by the mean value of variable importance for all of the taxa in descending order

**Fig. 5:**
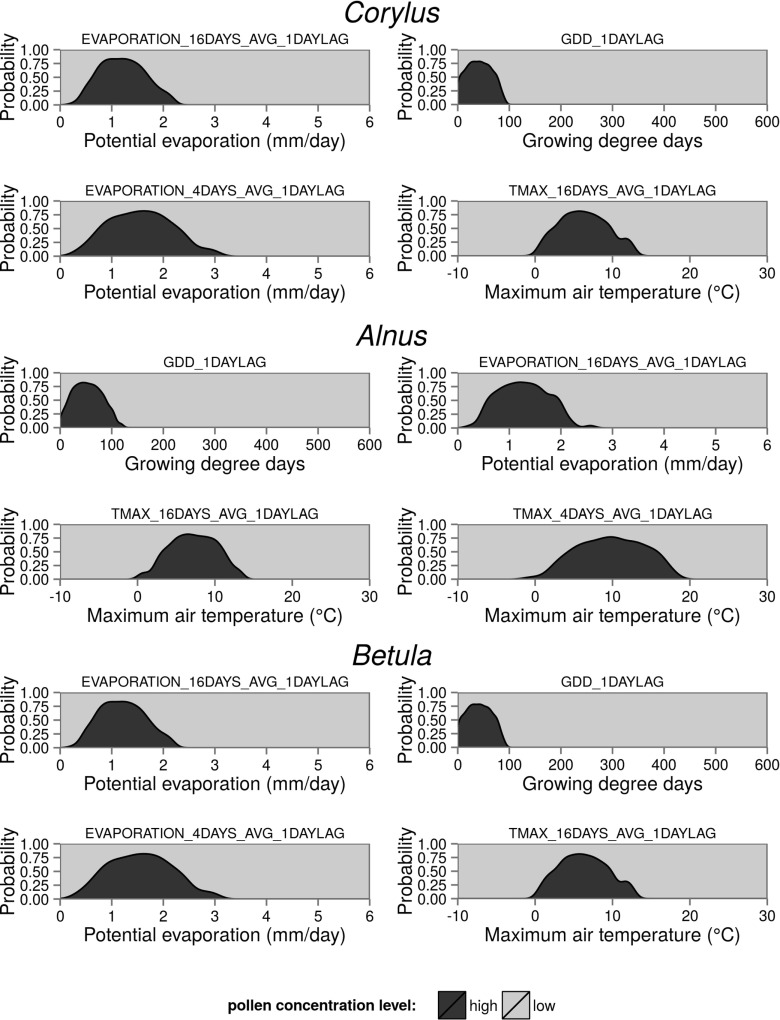
Relationship between probability of high pollen concentration level and the values of the four most important variables on the training set of the *Corylus*, *Alnus*, and *Betula* models

Using a random forest model, it is possible to examine predicted class probabilities for each variable in the dataset (Fig. 4). Figure 5 shows the relationship between the probability of high pollen concentration levels and the values of the four most important variables on the training set of the *Corylus*, *Alnus*, and *Betula* models. Cumulated growing degree days (GDD) was the most important variable in the *Alnus* and *Betula* models and the second-most important variable in the *Corylus* model. For each taxa, the highest probability of high pollen concentration level had a different range of GDD values. In the *Corylus* model, there was more than 0.5 probability of a high pollen concentration level when the GDD value was between 3 and 77. The highest probability (0.79) was connected with a GDD of 32. The range of probabilities and GDD values was slightly different in the *Alnus* model. A probability higher than 0.5 of high pollen concentration levels occurred when GDD was between 15 and 90. The peak of probability was 0.82 for a GDD value of 44. In the *Betula* model, the optimal value of GDD was between 94 and 323, with the highest peak (0.93) for a GDD of 183. Moreover, the distribution of high pollen concentration level probability in the *Betula* model was noticeably right-skewed. The 16-day average Penman potential evaporation from a free water surface also proved to be a highly important variable in all of the models. The optimal values of potential evaporation for predicting high pollen concentration levels were similar in the *Corylus* and *Alnus* models. In these models, potential evaporation values were between 0.6 and 1.7 (*Corylus*) and between 0.6 and 2 (*Alnus*) for at least 0.5 probability of high pollen concentration level. In the *Betula* model, optimal values of potential evaporation for high pollen concentration levels were between 1.7 and 3.6. In contrast, average monthly temperatures for the preceding year and spatial variables such as latitude, longitude, and altitude were the least important variables in the *Corylus*, *Alnus*, and *Betula* models (Fig. 4).

### Performance of the models

**Fig. 6:**
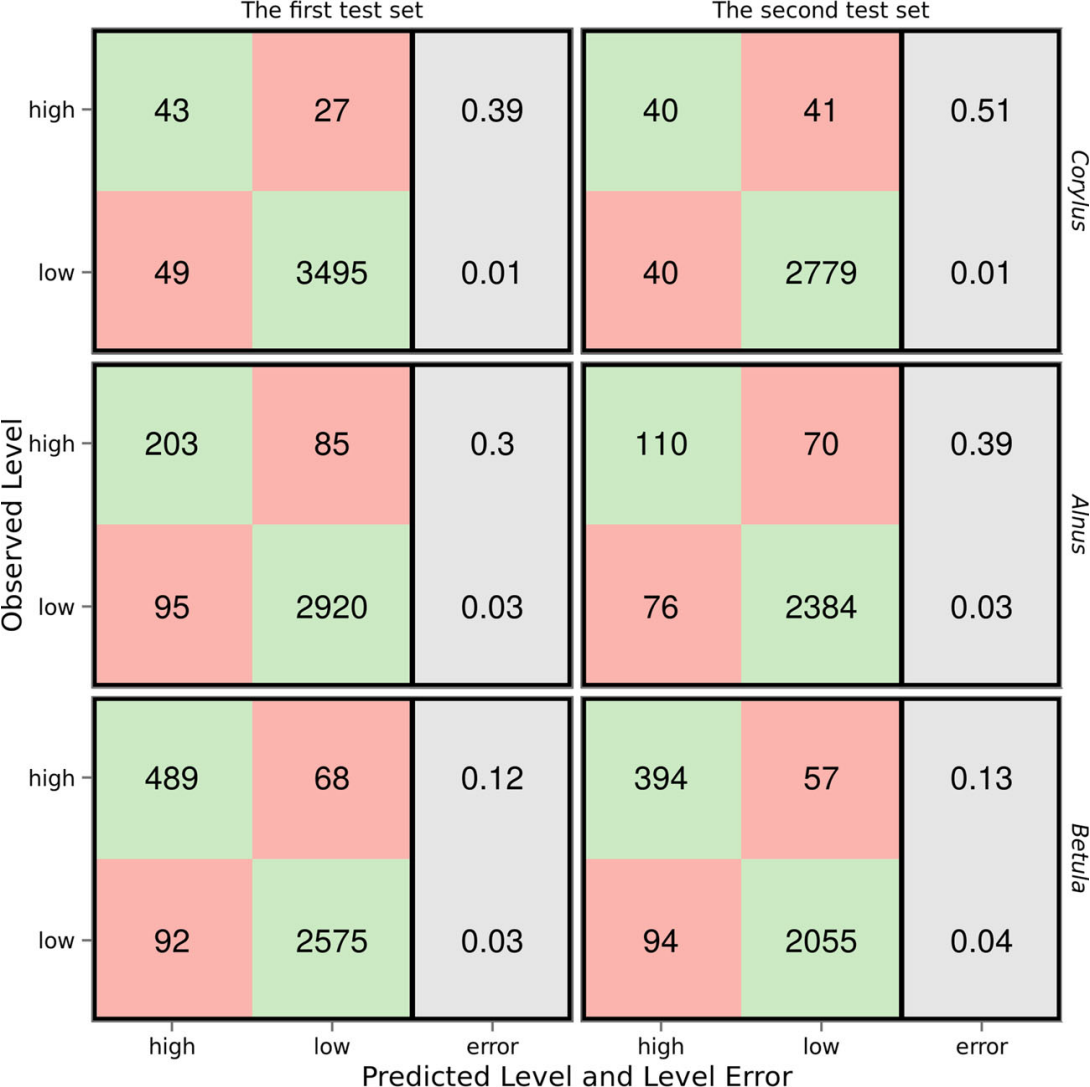
Relation between observed and predicted days with low and high concentration levels for *Corylus*, *Alnus*, and *Betula* pollen and prediction errors for the first and the second test set

The comparison of the models’ performance on the test sets showed several trends (Fig. 6). In general, the *Betula* model produced the best prediction on both test sets, while the *Corylus* model was the least accurate. Moreover, there was a difference between the quality of the temporal and spatial models. For each taxon, the model was better in predicting the pollen concentration level on the first test set. The differences between sensitivity and positive predictive value were visible between the first and second test set. All of the models were very accurate in predicting low pollen concentration levels. Specificity ranged between 0.96 for the *Betula* model on the second test set and 0.99 for the *Corylus* model on the first test set. The average model specificity was 0.97 for both the first and second test set.

#### Temporal performance of the models

The positive predictive value of the *Corylus* model prediction on the first training set was 0.47. However, it is more important to correctly predict high pollen concentration levels than to misclassify low pollen concentration levels. The *Corylus* model performed reasonably well in predicting high levels of pollen concentration, with a sensitivity of 0.61. The *Alnus* model correctly predicted 203 out of 288 occurrences of days with high pollen concentration (sensitivity = 0.70). Moreover, the *Alnus* model’s positive predictive value was distinctly higher than that of *Corylus*. The *Betula* model showed the best performance on the first test set. The model correctly classified approximately 88 % of days with high pollen concentration levels. The Kappa statistic value was 0.83, indicating a very high fit for the model.

#### Spatial performance of the models

The spatial quality was distinctly different in the models of each taxon. The *Corylus* model showed the lowest predictive capability. The model correctly predicted high pollen concentration levels in 40 out of 81 days (sensitivity = 0.49). The same model incorrectly classified 40 cases as high levels of pollen. The *Alnus* model performed better on the second test set. Both the models’ sensitivity and positive predictive value were clearly higher: 0.61 and 0.59, respectively. The *Alnus* model correctly predicted 110 occurrences of high pollen concentration levels and misclassified 76 cases as high level. On the second test set, the performance of the *Betula* model was found to be the best. The model Kappa statistic was 0.80, the sensitivity was 0.87, and the positive predictive value was 0.81. High *Betula* pollen concentration levels were correctly predicted in 394 of 451 cases. At the same time, only 94 days were incorrectly classified as high level.

### Predictive maps

**Fig. 7:**
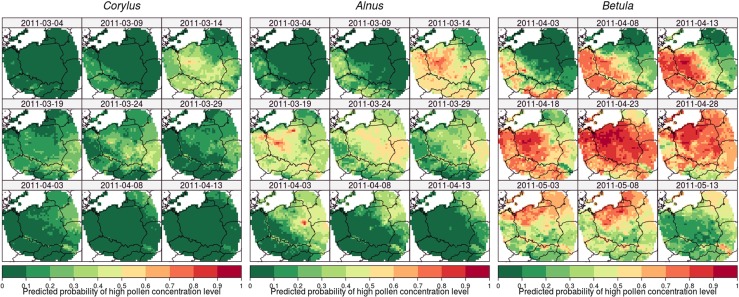
Examples of *Corylus*, *Alnus*, and *Betula* models’ prediction for nine regularly distributed days in the year 2011, based on the data for Poland and the area within 200 km of the Polish border

The *Corylus*, *Alnus*, and *Betula* models were built using processed variables from gridded meteorological data. Thus, it was possible to predict the probability of high pollen concentration levels for each cell and each date in the available data. Figure 7 shows examples of the *Corylus*, *Alnus*, and *Betula* models’ prediction for nine regularly distributed days in the year 2011.

## Discussion

*Corylus*, *Alnus*, and *Betula* pollen have an enormous impact on the quality of life and the productivity of allergy sufferers. Therefore, these tree pollen are the origin of significant social and financial burdens. Many aerobiological studies have been conducted in response to this problem, some of which have tried to build predictive models of pollen concentration (Bringfelt et al. 1982; Cotos-Yáñez et al. 2004; Castellano-Méndez et al. 2005; Rodriguez-Rajo et al. 2006; Vogel et al. 2008; Hilaire et al. 2012; Puc 2012; Sofiev et al. 2013). As a result of these studies, it is possible to predict high pollen concentration levels with considerable accuracy in the analyzed sites. However, in many countries, the aerobiological network is not dense, and therefore, it is not possible to predict pollen counts in unsampled locations. In this study, gridded meteorological data were used as predictor variables to build a model of high *Corylus*, *Alnus*, and *Betula* pollen concentration levels for spatially continuous areas of Poland.

The days with high pollen concentration levels of the analyzed taxa occur rarely. This property should be taken into consideration when building predictive models. This study used a novel technique of obtaining an optimal threshold, by minimizing the distance between sensitivity, specificity, positive predictive value, and negative predictive value and the best possible performance. Preliminary studies showed that, in this two-class problem, the optimizing probability threshold technique surpasses other strategies for overcoming class imbalances, such as upsampling and downsampling.

In the *Corylus*, *Alnus*, and *Betula* models, cumulated growing degree days lagged by 1 day proved to be one of the most important variables. The fully growth competent buds need stimulation before they can burst; therefore, the occurrence of temperatures above a certain base level is required (Dahl et al. 2013). Secondly, most of the 16-day averages of meteorological factors (daily potential evaporation, total global radiation, vapor pressure, minimum temperature, and maximum temperature) showed high values of the variable importance. Previous studies showed that the readiness to flower is dependent on, inter alia, light intensity, and evaporation (Pacini and Hesse 2004; Dahl et al. 2013). In contrast, the importance of the preceding years average monthly temperatures for each month and grid cell longitude, latitude, and altitude had little impact on the model. Although the studies of Latałowa et al. (2002) and Rasmussen (2002) found a relationship between the annual pollen sum and the mean temperature in the preceding year, this relationship has a small influence on the daily pollen concentration.

The *Corylus*, *Alnus*, and *Betula* models varied in terms of predictive quality. The *Corylus* model predicted correctly approximately 55 % of the high pollen concentration levels on the test sets. This model misclassification could be connected with very rare (330 cases, about 2.5 % of the analyzed days) occurrences of high *Corylus* pollen concentration levels. *Corylus* inflorescences produce about two times fewer pollen grains than *Alnus* inflorescences (Piotrowska 2008). Thus, a dataset of longer time periods or a denser monitoring network could result in a more precise model. The *Alnus* model performed better, with correct prediction of approximately 2/3 of high pollen concentration levels on the test sets. The problem of class imbalance was less severe in the *Alnus* dataset. Nonetheless, *Alnus* (and *Corylus*) pollen seasons are highly changeable from year to year. In addition, the location of aerobiological monitoring sites influences the variability of the pollen count of these taxa (Nowosad et al. 2015). The *Betula* model had the best values of model evaluation statistics. Almost 88 % of high pollen concentration levels were correctly predicted on the test sets. The negative impact of class imbalance was modest due to the relatively frequent occurrence of high *Betula* pollen concentration levels. Moreover, the *Betula* pollination period is relatively short and less changeable, and therefore, the *Betula* pollen count is more predictable.

The predictive quality of the *Betula* model is comparable to previous work. Castellano-Méndez et al. (2005) created a neutral network model for prediction of the risk of pollen concentration values exceeding a given level, using pollen and meteorological data. That model was built and validated in only one location: the city of Santiago, Spain. In contrast, in this study data from 11 aerobiological sites, as well as gridded meteorological data, were used in the process of model creation; therefore, model prediction should be verifiable in substantial areas surrounding the aerobiological monitoring sites.

Relationships between pollen concentration in the air and meteorological factors are complex and strongly nonlinear. Thus, classical statistical models, such as logistic regression or linear discriminant analysis, tend to perform poorly. Machine learning techniques could find patterns in nonlinear, noisy data, and generate prediction with relatively high accuracy (Recknagel 2001). Some of the most often used machine learning methods include nonlinear classification models (e.g., neural networks and support vector machines) and tree-based models (e.g., classification trees and random forest) (Kuhn and Johnson 2013). Random forest proved to give more accurate prediction than single tree models (Breiman 2001). In addition, this technique was compared to neural networks, and support vector machines require minimal preprocessing of the data. However, none of the single modeling techniques work best for every problem (Wolpert 1996). Therefore, it would be worthwhile to compare performance of different machine learning models for predicting pollen concentration.

Prediction errors of the *Corylus*, *Alnus*, and *Betula* models are the result of a combination of numerous factors: (i) omission of some non-meteorological predictors, (ii) influence of medium- and long-range pollen transport, and (iii) temporal and spatial uncertainty of pollen data. Meteorological conditions are also not the only factor that influence pollen concentration values. After the same meteorological conditions, high or low pollen concentration levels of *Corylus*, *Alnus*, and *Betula* can be observed on different occasions. Pollen concentration in the air is a result of nonlinear interactions between many factors, such as the land cover, topography, and human impact (Piotrowska and Kubik-Komar 2012). Taking into account the proportion of the analyzed taxa in the local vegetation could positively influence model quality. Previous studies also showed that most of recorded airborne pollen comes from local sources (Adams-Groom et al. 2002; Damialis et al. 2005). Nevertheless, medium- and long-range transport is also often recorded, as pollen grains are found hundreds or thousands of kilometers away from their source (Damialis et al. 2005; Ranta et al. 2006). In the *Corylus*, *Alnus*, and *Betula* models, the effect of long-range transport is not included. Moreover, uncertainty in the results of models could be connected with several characteristics of the data. The results of aerobiological monitoring are not the exact values of the pollen concentration of the surrounding area but are subject to errors from various sources, such as device, preparation of the sampling surface and slides, and slide analysis (Gottardini et al. 2009). In addition, there is diurnal variation in the number of pollen grains in the air (Galán et al. 1991; Skjoth et al. 2008). It is estimated that approximately 10 % of *Corylus*, *Alnus*, and *Betula* pollen count variations can be due to diurnal fluctuations and measurement errors (Nowosad et al. 2015). Only 11 aerobiological monitoring sites, which are not randomly distributed in Poland, were used in this study. The sites are located mainly in large cities, where the local climate is modified by human activities. These cities are significantly warmer than the surrounding rural areas, on average by 0.8–1.3 ^∘^C (Szymanowski 2005). Furthermore, the local airflow and turbulence are affected by buildings and non-building structures (Emberlin and Norris-Hill 1991). As a result, the deposition patterns in cities are different from those in the countryside (Emberlin and Norris-Hill 1991; Gonzalo-Garijo et al. 2006). Moreover, given the lack of sites in mountainous areas, caution should be exercised when using prediction models in those areas. In the long term, it will be valuable to add monitoring sites in remote rural areas, as well as in mountainous areas.

The modeling framework used in this study can be used as the basis for further research. The models are built based on meteorological factors and could be easily implemented in other countries. Moreover, it would be worthwhile to analyze the possibility of improving the models’ quality by utilizing non-meteorological parameters, such as the distribution of tree species and local land use.

## Conclusions

- In this study, the probability of high pollen concentration levels of *Corylus*, *Alnus*, and *Betula* was predicted using preprocessed gridded meteorological data. The result of the models could be used for prediction in continuous areas rather than just in single locations
- The models built allow moderately reliable predictions of high pollen concentration levels of *Corylus* and highly reliable predictions of high levels of *Alnus* and *Betula* pollen
- Temporal verifiability was higher than spatial verifiability in each of the *Corylus*, *Alnus*, and *Betula* models
- Average monthly temperatures for the preceding year were not very important for the results of the models
- Cumulated growing degree days was one of the most important variables in the *Corylus*, *Alnus*, and *Betula* models. In addition, sixteen-day averages of potential evaporation, total global radiation, vapor pressure, minimum temperature, and maximum temperature were important variables for the models.
- Spatial variables such as latitude, longitude, and altitude had little impact on the models
- The modeling framework could be applied in predicting high pollen concentrations of the different pollen taxa in the study sites and also in other areas
